# Genetic Variation of *Vibrio cholerae* during Outbreaks, Bangladesh, 2010–2011

**DOI:** 10.3201/eid2001.130796

**Published:** 2014-01

**Authors:** Shah M. Rashed, Andrew S. Azman, Munirul Alam, Shan Li, David A. Sack, J. Glenn Morris, Ira Longini, Abul Kasem Siddique, Anwarul Iqbal, Anwar Huq, Rita R. Colwell, R. Bradley Sack, O. Colin Stine

**Affiliations:** icddr,b, Dhaka, Bangladesh (S.M. Rashed, M. Alam, A.K. Siddique, A. Iqbal);; Johns Hopkins Bloomberg School of Public Health, Baltimore, Maryland, USA (A.S. Azman, D.A. Sack, R.B. Sack);; University of Maryland, Baltimore (S. Li, O.C. Stine);; University of Florida, Gainesville, Florida, USA (J.G. Morris, Jr., I. Longini);; University of Maryland, College Park, Maryland, USA (A. Huq, R.R. Colwell)

**Keywords:** multilocus sequence analysis, infectious disease outbreaks, Vibrio cholerae, bacteria, Bangladesh, cholera, outbreak

## Abstract

Cholera remains a major public health problem. To compare the relative contribution of strains from the environment with strains isolated from patients during outbreaks, we performed multilocus variable tandem repeat analyses on samples collected during the 2010 and 2011 outbreak seasons in 2 geographically distinct areas of Bangladesh. A total of 222 environmental and clinical isolates of *V. cholerae* O1 were systematically collected from Chhatak and Mathbaria. In Chhatak, 75 of 79 isolates were from the same clonal complex, in which extensive differentiation was found in a temporally consistent pattern of successive mutations at single loci. A total of 59 isolates were collected from 6 persons; most isolates from 1 person differed by sequential single-locus mutations. In Mathbaria, 60 of 84 isolates represented 2 separate clonal complexes. The small number of genetic lineages in isolates from patients, compared with those from the environment, is consistent with accelerated transmission of some strains among humans during an outbreak.

## Abstract 2

Most isolates are closely related, but genetic variation implies accelerated transmission of some lineages.

In many areas of the world, cholera remains a major public health problem; it affects millions of persons each year and causes a substantial number of deaths (*1*,*2*). In Bangladesh, cholera transmission is seasonal; 2 annual peaks are initiated by emergence of *Vibrio cholerae* from environmental reservoirs (*3*,*4*). The infectious dose of *V. cholerae* is estimated to be 10^5^ to 10^8^ CFU; the lower estimates are associated with a buffered stomach (*5*). After the organism enters the body, a physiologic change is induced, which alters the expression of most *V. cholerae* genes (*6*). One outcome of this alteration is a brief hyperinfectious state, during which *V. cholerae* exiting the colon are infectious at a reduced dose (*7*). After returning to the water for 24 hours, *V. cholerae* reverts to a standard infectious state (*7*,*8*). The relative contribution of the recently shed hyperinfectious strains and the strains from the environment to propagation of an outbreak of cholera remains controversial. The terms “slow” and “fast” have been used to distinguish between these 2 modes of transmission; slow refers to the human-to–aquatic environment–to-human pathway (which does not have time constraints), and fast refers to presumed person-to-person or person-to–household environment–to-person transmission (in which transfer is more likely immediately after fecal shedding, when strains are hyperinfectious) (*9*).

To determine the relative contribution of the hypothesized slow and fast routes of transmission during outbreaks, researchers have undertaken microbiological, genetic, and modeling approaches. However, a major problem with the first 2 approaches has been a lack of genetic diversity to track strains. Many methods, including pulsed-field gel electrophoresis (often used for outbreak analysis), detect too few genetic differences between isolates to be useful in tracking the microdynamics of strains. This problem was resolved, in part, by identification of loci containing a variable number of tandem repeats, which provided enough genetic variability to permit tracking of specific strains (*10*–*12*). However, initial studies that used multilocus variable tandem repeat analysis (MLVA) did not sample intensively enough to optimally distinguish between slow and fast transmission. One study, conducted in rural Bangladesh, in which isolates were collected every 2 weeks, showed that isolates from different geographic locations collected during different seasons and from clinical and environmental sources had only a few genotypes in common (*12*). Genotypes tended to be similar to one another when isolates were collected during the same season and came from the same geographic location as opposed to coming from different seasons or sources. Although this finding could be interpreted as evidence in support of fast transmission, most environmental isolates were not collected during the same month as most of the clinical isolates, making interpretation of data difficult. In another study, isolates from 100 index case-patients and their household relatives were analyzed (*13*). Remarkably, isolates from persons within a single household were often genetically unrelated, implying either different sources of infection or a single source with multiple genetic lineages. The unexpected variability was reinforced by the observation that unrelated genetic isolates were often isolated from a single fecal sample. However, the study design of sampling 3 households per month did not provide sufficient resolution to address transmission pathways. Mathematical modeling of incidence data from outbreaks has been used to estimate the contribution of fast and slow transmission (*14*,*15*). Although promising, these estimates have been based on clinical surveillance data without more detailed underlying epidemiologic information. Furthermore, it might not be possible to estimate the contribution of 2 transmission mechanisms from incidence data alone when the time scale of slow transmission is similar to that of fast transmission (*16*).

In this study, we used MLVA to characterize 222 clinical and environmental *V. cholerae* O1 isolates from outbreaks in 2 geographically distinct locations. Our objective was to determine genetic relatedness among the isolates, notably clonal relationships between environmental and clinical isolates, and to further explore the relative contribution of different transmission pathways to disease occurrence.

## Materials and Methods

### Sample Collection

From October 2010 through May 2011, clinical and environmental samples were collected in Chhatak and Mathbaria, Bangladesh. Chhatak is a village located 180 km northeast of Dhaka in the central region, and Mathbaria is a rural area located 150 km south-southeast of Dhaka in the coastal region. 

Clinical isolates were obtained from rectal swabs from all patients who visited a health center with signs or symptoms of suspected cholera, representing ≈40% of all suspected cases per week during the peak season of cholera. Clinical samples were collected during 3 consecutive days every week. For characterization of within-host variability of *V. cholerae*, 9–10 isolates were selected from a culture from each of 6 patients at the Chhatak clinic. 

Environmental isolates were obtained from water and plankton samples collected at 6 sites (ponds or rivers) in each community, as described (*17*). The same environmental sites were sampled, and the same sampling methods were used each time. Water and plankton samples were collected, concentrated, and analyzed for *V. cholerae* according to standard procedures (*18*,*19*). *V. cholerae* isolation and identification were performed according to standard methods (*19*,*20*). DNA was prepared from broth cultures of *V. cholerae* by using PrepMan Ultra (Applied Biosystems, Foster City, CA, USA) according to the manufacturer’s instructions. All samples were collected according to protocols approved by institutional review boards at Johns Hopkins University, University of Maryland (both in Baltimore, MD, USA), and icddr,b (Dhaka, Bangladesh).

### Genetic Analysis

All PCRs were conducted by the same technician. Fluorescently labeled PCR primers were used to amplify 5 loci with variable-length tandem repeats; a new reverse primer was used for VCA0283 (5′-AGCCTCCTCAGAAGTTGAG-3′) (*13*). The reaction products were separated, detected, and sized by using a 3730xl automatic sequencer, internal lane standards (Liz600), and GeneScan program (all from Applied Biosystems). Genotypes were determined by using published formulas to calculate the number of repeats from the length of each allele except for VC0437, which is calculated by (*x* – 252)/7 (*13*). Five loci were ordered: VC0147, VC0437, VC1650, VCA0171, and VCA0283. A genotype (e.g., 9-4-6-19-11) was interpreted as an isolate having alleles of 9, 4, 6, 19, and 11 repeats at the 5 loci, respectively. Relatedness of isolates was assessed by using eBURST version 3 (http://eburst.mlst.net). Genetically related genotypes are defined as those possessing identical alleles at 4 of the 5 loci and groups of related genotypes (clonal complexes). Additional sensitivity analyses were conducted by using only the 3 more stable loci from the large chromosome (VC0147, VC0437, VC1650); relatedness was determined by identical alleles at 2 of the 3 loci (*13*,*21*).

### Statistical Analyses

The null hypothesis was that the genotype isolated from each clinical case has an equal probability of being from each of the genetic lineages (for 5-locus analysis, from 1 of the observed clonal complexes or singletons; for 3-locus analysis, the same genotype) from the environment (*p_1_ = p_2_ = … = p_n_*), which, if true, would be evidence in support of a strong role of the slow transmission route in these outbreaks. A χ^2^ test was used to determine whether the null hypothesis was true. The Simpson index was calculated as a measure of genotype diversity (low values indicating more diversity) within each location as

 where is the total number of genotypes at a particular location and is the proportion of genotypes of type . 

## Results

During the study period, 222 *V. cholerae* O1 Ogawa isolates were obtained from clinical and environmental samples from both locations. In Chhatak, fecal samples from 74 patients yielded 1 isolate from each patient; 6 samples yielded 9–10 additional isolates each. The environmental samples from 6 ponds yielded 5 isolates. In Mathbaria, fecal samples from 56 patients yielded 1 isolate each; the environmental samples from 6 ponds yielded 28 isolates. A total of 51 five-locus genotypes were observed, of which only 3 genotypes were from both Mathbaria and Chhatak, a result consistent with prior observation of geographic differences (*12*). Thus, isolates from the 2 locations were analyzed separately.

### Chhatak

Extensive genetic variation was detected among the 138 isolates (Table 1, Simpson index 0.10). The numbers of distinct alleles at loci VC0147, VC0437, VC1650, VCA0171, and VCA0283 were 6, 3, 1, 12, and 8, respectively, with a minimum of 4 and up to 27 repeat units. A total of 30 genotypes (each a unique combination of alleles at the 5 loci) were detected. Only 1 genotype was found in both clinical and environmental isolates. An additional 26 genotypes were found among the clinical isolates, and an additional 3 were found among the environmental isolates.

**Table 1:** Genotypes of *Vibrio cholerae* isolated from Chhatak, Bangladesh, October 2010–May 2011

Genotype	Source	No. isolates* (1/sample)	No. isolates† (9–10/sample)	Clonal complex
8-4-14-21-18	Human	7		1
8-4-14-22-18	Human	1		1
9-4-14-14-16	Human	8		1
9-4-14-14-17	Human	1		1
9-4-14-9-17	Human	1		1
9-4-14-20-17	Human	2		1
9-4-14-21-12	Human	11		1
9-4-14-21-13	Human	1		1
9-4-14-21-19	Human	1		1
9-4-14-21-18	Human	5	4	1
9-4-14-22-12	Human	2		1
9-4-14-22-16	Human	2		1
9-4-14-23-16	Human	1	1	1
9-4-14-23-17	Human	1	3	1
9-4-14-23-18	Human	5		1
9-4-14-23-19	Human	1		1
9-4-14-25-16	Human	1		1
9-4-14-25-17	Human	15	14	1
9-4-14-21-17‡	Human and environment	5 and 2	18	1
9-4-14-27-18	Environment	1		1
8-4-14-21-11	Environment	1		1
7-4-14-23-17	Human		5	1
9-4-14-13-16	Human		1	1
9-4-14-18-16	Human		9	1
9-4-14-22-18	Human		2	1
9-4-14-22-17	Human		1	1
7-9-14-14-6	Human		1	Singleton, Oct 13
11-9-14-14-17	Human	2		Singleton, Oct 21
12-8-14-15-17	Human	1		Singleton, Oct 11
10-8-14-17-18	Environment	1		Singleton, Nov 1

*Single isolates chosen from the 79 samples **†**Multiple isolates from 6 patients ‡Designates the founder genotype defined by eBURST (http://eburst.mlst.net) as the genotype that differs from the largest number of genotypes at a single locus.

**Figure 1:**
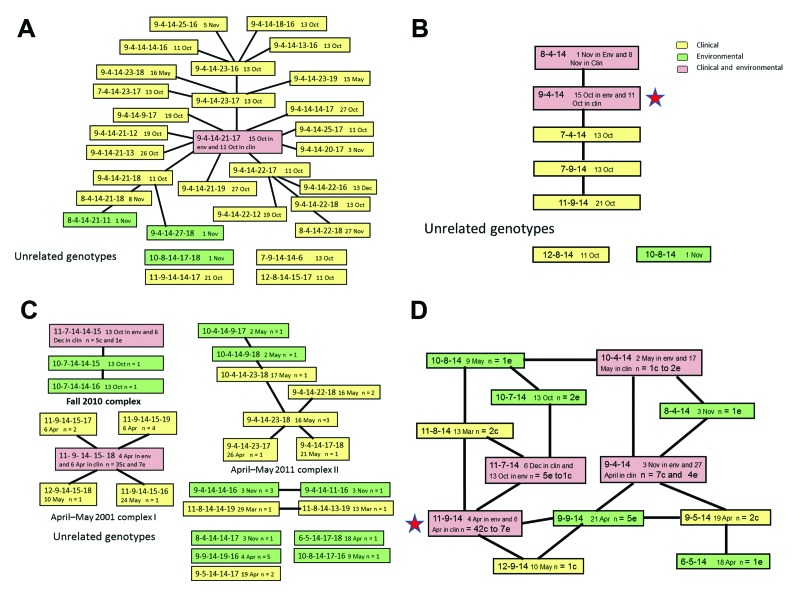
Genetic relatedness between *Vibrio cholerae* genotypes, Bangladesh, 2010–2011. Each genotype is identified by the number of repeats in the allele at the 5 loci VC0147, VC0437, VC1650, VCA0171, and VCA0283. The earliest date of detection is recorded in the box after the fifth allele. The background of the box indicates whether the genotype was detected in clinical isolates only (yellow), environmental isolates only (green), or both (pink). A) Clonal complex of genotypes from Chhatak, Bangladesh, and 5 unrelated genotypes based on a 5-locus genotype. B) Analysis of 3-locus genotypes in Chhatak. C) Five clonal complexes of isolates from Mathbaria, Bangladesh, and 4 unrelated genotypes based on a 5-locus genotype. D) Analysis of 3-locus genotypes from Mathbaria. Env, environmental; clin, clinical.

Analysis of the genotypes by eBURST showed that 26 genotypes clustered into 1 clonal complex (Figure 1, panel A), and 4 genotypes were singletons. The founder of a clonal complex is defined as the genotype with the largest number of single-locus variants (SLVs). In this clonal complex, the founder genotype 9-4-14-21-17 was present in 7 clinical and 2 environmental isolates. The 10 SLV genotypes diverging from the founder genotype were all clinical isolates. Of these 10 SLV genotypes, 3 (9-4-14-23-17; 9-4-14-22-17; 9-4-14-21-18) differentiated further into 9 double-locus variants. Of these, 3 double-locus variant genotypes were differentiated even further into 6 additional variants, often a variant of a locus already noted as a variant.

**Figure 2:**
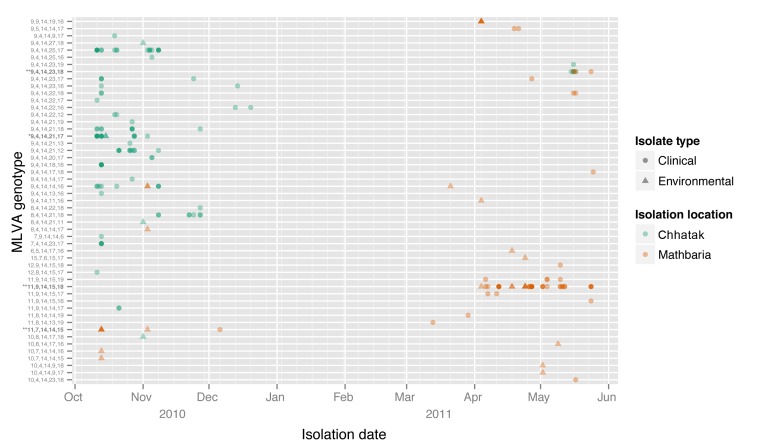
Day of *Vibrio cholerae* isolation and genotype, Bangladesh, 2010–2011. Multiple genetically identical isolates on the same day increase the darkness of the shape. MLVA, multilocus variable tandem repeat analysis.

In any outbreak, time is a critical variable for the temporal sequence of isolations (the earliest date of detection is incorporated into Figure 1 and Figure 2, Appendix). The founder, and 4 related genotypes, were isolated from multiple cases on the first day, October 11, 2010. Thereafter, on October 13, an additional 6 related genotypes were isolated. The founder was recovered from the environment on October 15. The 10 SLVs were collected from October 11 through November 3. Of the 25 variants, 23 were isolated on the same day or after the genotype next closest to the founder, which is the expected result if the genotypes are derived successively from the founder. If instead of evolving during the outbreak, each genotype existed initially in the environment, then we would expect the probability of finding related genotypes before versus after the founder (or next closest to the founder) to be 0.50. However, we observed 23 of 25 after the founder (or next closest) and, if derived from a random (binomial) process, we would expect to see >23 with probability of 10**^−5^**. Among the genotypes distant from the founder, only 2 were isolated from the environment, on November 1 and November 8, postdating the first observation of the SLV genotype closer to the founder, which was from a clinical isolate.

Most of the variation occurs in the 2 loci (the fourth and fifth of the genotype) on the small chromosome. If we analyze only the more stable loci, each isolate is described by a 3-locus genotype, and only 7 genotypes are observed. Five genotypes could be related to another genotype by a mutation at 1 locus (Figure 1, panel B), and 2 genotypes were unrelated. The 9-4-14 genotype occurred in 86% (64/74) of the clinical cases sampled.

Variation among isolates from a single fecal sample were also observed (Table 2). Of the 6 fecal samples from which multiple isolates were tested, only 1 yielded 1 genotype for all isolates, 2 yielded 2 genotypes, 2 yielded 3 genotypes, and 1 yielded 4 genotypes. For 4 of 5 fecal samples, all genotypes of the isolates were related variants; variations were seen only in loci of the small chromosome. In 1 sample, 1 isolate was unrelated to any of the other 3 genotypes observed in the other 9 isolates, evidence of multiple isolates involved in disease production. Additional evidence for multiple isolates being involved in disease is provided by the observation that 5 of the 6 genetic lineages in Chhatak were found in clinical samples.

**Table 2:** Genotypes of the 59 *Vibrio cholerae* isolates from 6 fecal samples from cholera patients, Chhatak, Bangladesh, 2010*

Patient	Collection date	No. isolates	Genotypes	Number	Relation to founder†
1	Oct 11	10	9-4-14-**25**-17	10	SLV
2	Oct 11	9	9-4-14-21-17	5	Founder
			9-4-14-**25**-17	4	SLV
3	Oct 11	10	9-4-14-21-17	7	Founder
			9-4-14-21-**18**	2	SLV
			9-4-14-**22**-17	1	SLV
4	Oct 13	10	**7-**4-14-**23**-17	5	DLV
			9-4-14-**23**-17	3	SLV
			9-4-14-**23-16**	1	DLV
			7-9-14-14-6	1	Unrelated
5	Oct 13	10	9-4-14-**18-16**	9	DLV
			9-4-14-**13-16**	1	DLV
6	Oct 13	10	9-4-14-21-17	6	Founder
			9-4-14-21-**18**	2	SLV
			9-4-14-**22-18**	2	DLV

*9 or 10 isolates/sample. Variant alleles from founder genotype are in **boldface.** †SLV, single-locus variant; DLV, double-locus variant.

The genetic diversity observed in these environmental and clinical isolates enables crude inference about whether there is >1 mode of transmission. If all genotypes found in the clinical isolates were randomly sampled from the 5 genetic lineages of the 5-locus genotypes, or from 1 of the 7 three-locus genotypes, this would provide evidence for a strong role for slow transmission. However, we observed that 1 lineage predominates (p**<**10**^−5^**, χ^2^ test, 5-locus set; p**<**10**^−5^**, χ^2^ test, 3-locus set) consistent with the presence of a nonrandom mode of transmission among patients in Chhatak.

### Mathbaria

Significant genetic variation was also observed among the 84 isolates collected in Mathbaria (Table 3, Simpson index = 0.26). The numbers of distinct alleles at loci VC0147, VC0437, VC1650, VCA0171, and VCA0283 were 6, 5, 1, 8, 5, respectively. A total of 24 genotypes were detected, of which 14 were in 56 clinical isolates, 12 in 28 environmental isolates, and 2 in both types of isolate. Almost twice as many genetic lineages detected in Chhatak were detected in Mathbaria: 5 clonal complexes and 5 singletons. Two clonal complexes comprised only 2 genotypes. Among the unrelated (singleton) genotypes, 4 were found only in environmental isolates and 1 was found in a clinical isolate.

**Table 3:** Genotypes of *Vibrio cholerae* isolated from Mathbaria, Bangladesh, October 2010–May 2011

Genotypes	Source	No. isolates	Clonal complex
9-4-14-23-18*	Human	3	1
10-4-14-23-18	Human	1	1
10-4-14-9-18	Environment	1	1
9-4-14-17-18	Human	1	1
9-4-14-22-18	Human	2	1
9-4-14-23-17	Human	1	1
10-4-14-9-17	Environment	1	1
11-9-14-15-18*	Human and environment	35 and 7	2
11-9-14-15-17	Human	2	2
11-9-14-15-16	Human	1	2
11-9-14-15-19	Human	4	2
12-9-14-15-18	Human	1	2
11-7-14-14-15	Human and environment	1 and 5	3
10-7-14-14-15*	Environment	1	3
10-7-14-14-16	Environment	1	3
9-4-14-14-16	Environment	3	4
9-4-14-11-16	Environment	1	4
11-8-14-14-19	Human	1	5
11-8-14-13-19	Human	1	5
9-5-14-14-17	Human	2	Singleton, Apr 19
6-5-14-17-18	Environment	1	Singleton, Apr 18
8-4-14-14-17	Environment	1	Singleton, Nov 3
9-9-14-19-16	Environment	5	Singleton, Apr 4
10-8-14-17-16	Environment	1	Singleton, May 9

*Founder genotype defined as the genotype that has the most single-locus variants.

In Mathbaria, *V. cholerae* O1 isolates were obtained during the last 3 months of 2010 and during an outbreak during March–May 2011. In 2010, the clonal complex comprised 3 genotypes (Figure 1, panel C). All 3 were in environmental isolates before identification in an isolate from a clinical sample in December. The founder genotype was 1 of the 2 genotypes isolated from the environment on October 13. A second clonal complex of 2 genotypes and an isolate with an unrelated genotype were also collected from the environment at that time.

The annual (seasonal) outbreak of cholera in Mathbaria occurred during March–May 2011; during this outbreak, 3 distinct clonal complexes and 4 unrelated genotypes were detected. The smallest clonal complex was detected in 2 clinical isolates in March. In the largest clonal complex (April–May complex I in Figure 1, panel C), the founder genotype was observed in isolates from the environment on April 4. On April 6, the founder genotype and 2 others were identified in clinical isolates. Over the next 48 days, the founder genotype was detected in 42 isolates (35 from clinical patients and 7 from the environment). In May 2011, another 2 genotypes from this clonal complex were found. The intermediate size clonal complex (April–May complex II in Figure 1, panel C) was first observed in an isolate from a patient on April 26 and subsequently in 2 isolates from the environment on May 2. The founder genotype was observed in 3 clinical isolates on May 16. Subsequently, 3 other SLV genotypes were observed in clinical isolates. A clear pattern of the founder being detected initially and supposedly derived genotypes being detected later was not found for this clonal complex.

When the genotypes were defined only by the 3 large chromosome loci, 12 genotypes were observed, each related to >1 of the other genotypes. As shown in Figure 1, panel D, a network best describes the relatedness because no a priori method exists by which to assign which mutation is more likely to have happened. Two genotypes varied by 1 mutation from 4 other genotypes, and 6 genotypes varied by 1 mutation from 3 other genotypes. In the seasonal outbreak, the 11-9-14 genotype was observed in 42 (76%) of 55 of the isolates.

The genetic lineages observed in the clinical isolates were a nonrandom sample of those found in Mathbaria. Although 5 the 10 lineages were from clinical isolates, 1 lineage was found in 43 (77%) of 56 isolates. Assuming that any genetic lineage could cause a clinical case of disease with equal probability, then the observed results are nonrandom (p<10**^−5^**, χ^2^ test, 5-locus set). For the 3-locus analysis, there were 12 genotypes, and 1 occurred in 42 of 56 clinical isolates, a significantly nonrandom distribution (p<10**^−5^**, χ^2^ test, 3-locus set) and, similar to Chhatak, an accelerated mode of transmission (i.e., human-to-human) is likely for certain genotypes.

## Discussion

The results of this study show that multiple genetic lineages of *V. cholerae* occur naturally in the environment with geographic and seasonal genetic variation. The genotype patterns of the environmental and clinical isolates in the 2 rural Bangladesh communities indicate 2 things: that identical genotypes can be found in the environment and humans and that the genotypes in humans are not a random sample of those in the environment. The result of a simple χ^2^ test provides evidence that an accelerated human-to-human mode probably contributed to a process that generates genetic uniformity among clinical isolates. Whether the accelerated mode incorporates the hyperinfective state or involves massive numerical increases of a genetic lineage from the earliest cases cannot be distinguished from our analyses.

The rapid expansion of *V. cholerae* in Chhatak fits the pattern of a founder flush event (*22*). The founder flush principle asserts that rapid expansion of population size can be accompanied by relaxation of selection pressure so that genotypes otherwise not detected might be observed. This principle was applied to the presence of novel multilocus sequence genotypes in *V. cholerae* O139 (*23*). In Chhatak, after no clinical isolates were recovered in September and early October, on October 11 and 13, another 7 patients visited the clinic and 11 genotypes were observed among the isolates obtained from rectal swab samples. The expansion continued; over the next 26 days, 50 cases yielded 11 more genotypes. During the spring outbreak in Mathbaria, 2 clonal complexes exhibited additional genetic differentiation but not as dramatic as that in Chhatak. However, the outbreak in Mathbaria was smaller than that in Chhatak.

The appearance of novel MLVA genotypes that differ from the founder over time, as occurred in the Chhatak outbreak, is a microcosm of the evolution seen previously. In Kolkata, India, among *V. cholerae* O139 isolates, the founder genotype appeared shortly after the initial mutation from O1 to O139 and then mutated into multiple novel genotypes; mutations continued over the course of several years (*11*). Similarly, in Dhaka, isolates O1 Ogawa, O1 Inaba, and O139 mutated, and a clear progression of genotypes was documented from year to year (*13*). Previous studies found that differentiation occurred over several years, but in Chhatak, the differentiation occurred within 3 months.

Our analyses of the 3 first chromosome loci demonstrate that the outbreak has a dominant genotype drawn from many in the area. These large (first) chromosome loci are considered to be more stable than the small (second) chromosome loci (*13*,*21*). Although the second chromosome loci might be less useful in the context of evolution across decades (*21*), in this context, the increased resolution of local genetic linages in Chhatak reveals that many genetic variants might occur during rapid expansion, although increased variation is not an obligate part of the expansion, as indicated by the data from Mathbaria.

Multiple genotypes of *V. cholerae* were isolated from single fecal samples, as reported (*13*). However, unlike previous reports, in which only a minority (3 of 9) of samples contained isolates differing only in successive single allelic changes, in the study reported here, such samples accounted for a majority (5 of 6). Thus, whether the different genotypes are part of the same inoculum or differentiated during infection is impossible to determine. In the earlier study, 6 of the 9 fecal samples yielded isolates with unrelated genotypes (i.e., different clonal complexes). The study reported here was conducted in a rural area. Also, the clinical samples analyzed were collected during a seasonal outbreak, as opposed to ongoing infections throughout the year. Most isolates from patients in the surrounding community were of the same clonal complex; in the previous study, they were not.

Our results provide evidence in support of an accelerated mode of transmission and for multiple strains comprising an infective dose for cholera. As shown by the clinical isolates, a single isolate does not sufficiently describe a single clinical sample; this observation should be included in future clinical studies.
